# Supplementary material for: Development of ISB 1442, a CD38 and CD47 bispecific biparatopic antibody innate cell modulator for the treatment of multiple myeloma
Source: Nat Commun. 2024 Mar 6;15:2054. doi: 10.1038/s41467-024-46310-y (PMC10917784; doi:10.1038/s41467-024-46310-y)
Supplement: Supplementary file 1 — Supplementary Information [file 41467_2024_46310_MOESM1_ESM.pdf]

Supplementary Information File

Development of ISB 1442, a CD38 and CD47 bispecific biparatopic antibody innate cell modulator for the treatment of multiple myeloma

C. Grandclément [#,1], C. Estoppey [#,1], E. Dheilly [#,1], M. Panagopoulou [1], T. Monney [1], C. Dreyfus [1], J. Loyau [1], V. Labanca [1], A. Drake [1], S. De Angelis [1], A. Rubod [1], J. Frei [1], L. N. Caro [1], S. Blein [1], E. Martini [1], M. Chimen [1], T. Matthes [2], Z. Kaya [3], C. M. Edwards [3], J. R. Edwards [3], E. Menoret [4], C. Kervoelen [4], C. Pellat-Deceunynck [4,5], P. Moreau [4,5,6], M.L. Mbow [1], A. Srivastava [1], M.R. Dyson [1], E.A. Zhukovsky [1], M. Perro [1,#,*] and S. Sammicheli [1,#,*]

# Equally Contributing Authors, * Corresponding authors

[1] *Ichnos Glenmark Innovation, Lausanne, CH*
[2] *Haematology Service, Department of Oncology and Clinical Pathology Service, Department of Diagnostics, University Hospital Geneva, 1211, Geneva, Switzerland.*
[3]*Nuffield Department of Orthopaedics, Rheumatology and Musculoskeletal Sciences, Botnar Institute, University of Oxford, Oxford, United Kingdom.*
[4]*Nantes Université, Inserm, CNRS, Université d'Angers, CRCI2NA, Nantes, France*
[5]*SIRIC ILIAD, Angers, Nantes, France*
[6]*Service d'Hématologie Clinique, Unité d'Investigation Clinique, CHU, Nantes, France.*

Supplementary Figure 1: Affinity measurements of ISB 1442 for CD38 and CD47 by Surface Plasmon Resonance

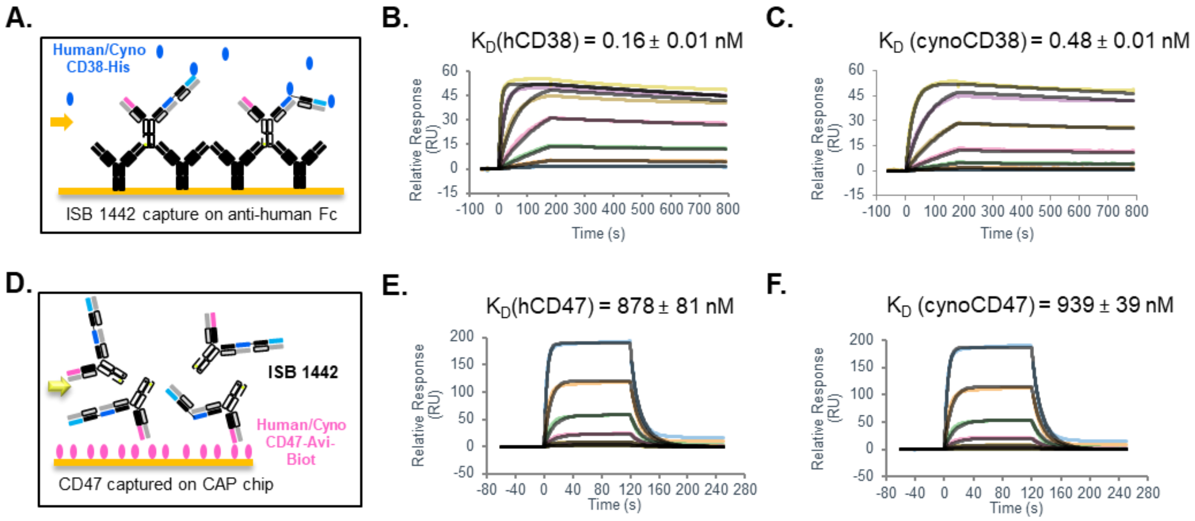

Supplementary Figure 1: Affinity measurements of ISB 1442 for CD38 and CD47 by Surface Plasmon Resonance

A. Schematic view of the assay setup for measurement of ISB 1442 affinity for CD38. ISB 1442 is immobilized on anti-human Fc chip and human or cynomolgus monkey CD38 is flushed onto the immobilized antibody at increasing concentrations. B-C. Fitted sensorgrams of a representative measurement show the binding of human (B) and cynomolgus monkey (C) CD38 onto immobilized ISB 1442 at increasing concentrations ranging from 0.14 nM to 100nM. Colored curves represent experimental data and black curves represent 1:1 kinetic fit. Average K_D (± standard deviation) of at least three replicates is shown. D. Schematic view of the assay setup for measurement of ISB 1442 affinity for CD47. Biotinylated CD47 is immobilized on CAP chip and ISB 1442 is flushed onto the immobilized CD47 at increasing concentrations. E-F. Fitted sensorgrams of a representative measurement show the binding of ISB 1442 onto immobilized human (E) and cynomolgus monkey (F) CD47 at increasing concentrations ranging from 2.7 nM to 2 µM. Colored curves represent experimental data and black curves represent 1:1 kinetic fit. Average K_D (± standard deviation) of at least three replicates is shown.

Supplementary Figure 2. Competition anti-CD38-B6-D9 Fab and anti-CD38-E2RecA Fab to isatuximab

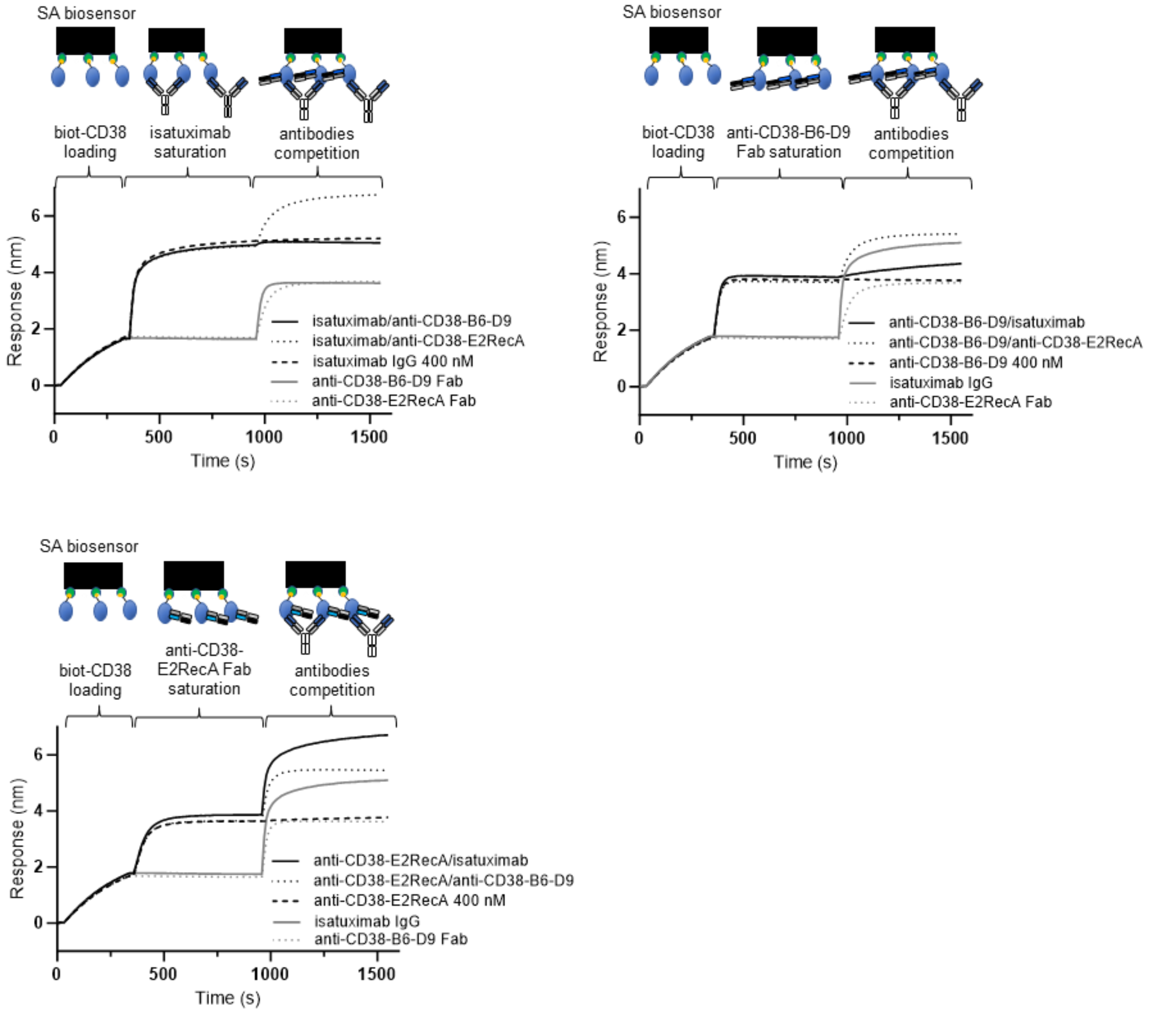

Supplementary Figure 2: Competition anti-CD38-B6-D9 Fab and anti-CD38-E2RecA Fab to isatuximab

Competition binding assay by Octet Bio-Layer Interferometry (BLI) between anti-CD38-B6-D9 Fab, anti-CD38-E2RecA Fab and isatuximab for binding to CD38. CD38-loaded biosensor was dipped in a solution of isatuximab (top left panel), anti-CD38-B6-D9 Fab (top right panel) or anti-CD38-E2RecA Fab (lower left panel) to reach saturation. Then, saturated biosensor was dipped into a premixed solution of saturating Fab and a second Fab (competition phase). Saturation of the CD38 sensor surface was verified by dipping antibody-saturated CD38 sensor surface into a solution of the same antibody at 2-fold concentration of saturation solution (400 nM). Grey lines represent controls to assess maximum binding of the second antibody in the absence of the first antibody. Plots show binding to the sensor tip as a wavelength shift (Response) over time. Curves are labelled by antibodies solution in competition phase.

Supplementary Fig 3: Imaging of phagocytosis of CD38^{low} Multiple Myeloma cells

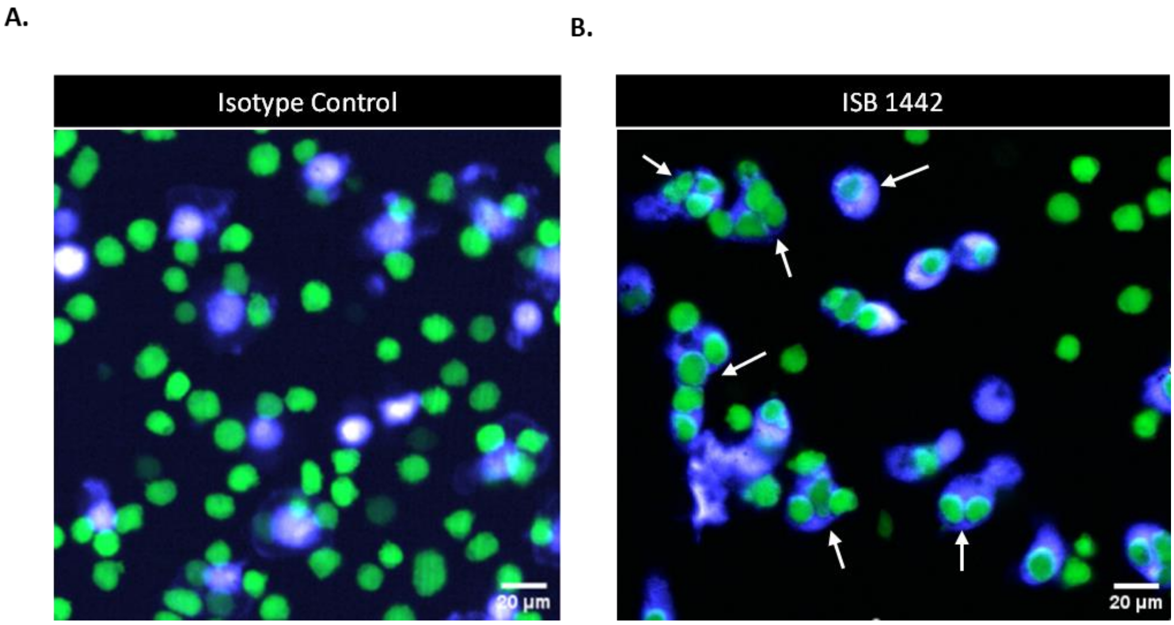

Supplementary Figure 2: Live imaging performed with Perkin Elmer Operetta in the presence of control antibody or ISB1442 used at 80nM. Snapshots from supplementary movies 1 and 2 showing monocytes-derived macrophages (blue) engulfing of KMS-12-BM CD38^{low} tumor cells (green). Arrows show examples of cells phagocytosis.

Supplementary Figure 4. Competition with daratumumab

A.

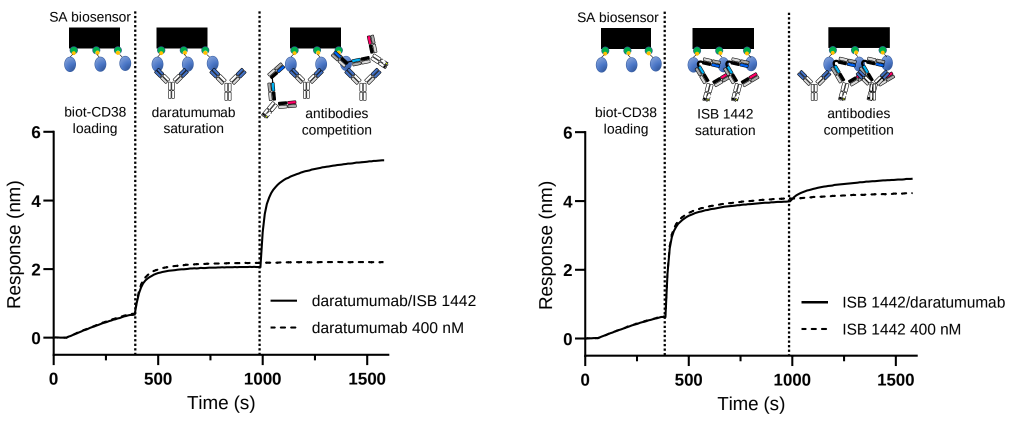

Ab ID	ISB 1442	Daratumumab
ISB 1442		38
Daratumumab	91	

B.

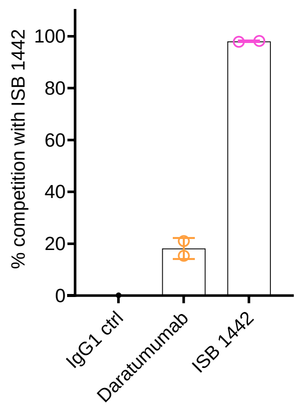

Supplementary Figure 4: Competition with daratumumab

A. Competition binding assay by Octet Bio-Layer Interferometry (BLI) between ISB 1442 and daratumumab for binding to CD38. CD38-loaded biosensor was dipped in a solution of daratumumab (top left panel) or ISB 1442 (top right panel) to reach saturation. Then, saturated biosensor was dipped into a premixed solution of saturating antibody and the second antibody (competition phase). Saturation of the CD38 sensor surface was verified by dipping antibody-saturated CD38 sensor surface into a solution of the same antibody at 2-fold concentration of saturation solution (dashed lines). Plots show binding to the sensor tip as a wavelength shift (Response) over time. Curves are labelled by antibodies solution in competition phase. Lower left panel: Plot of saturating antibody in rows against competing antibodies in columns. Values indicate percentage of binding of

competing antibody to CD38 relative to maximum binding in the absence of saturating antibody. Values less than 30 were assigned as competing antibodies and shaded in black, values between 30 and 70 were assigned as partially competing antibodies and shaded in grey, while values greater than 70 were assigned as non-competing antibodies. Self-blocks are outlined by a dark-grey box. B. Percentage of competition with ISB 1442 mediated by control antibody (IgG1 ctrl), daratumumab (AF647 conjugated) or ISB 1442 (AF488 conjugated) antibodies in Raji-CD47KO cells. Mean of technical duplicates is represented here +/- Standard Deviations (SD). N=2.

Supplementary Figure 5. ISB 1442 binding on leukemia cell lines in comparison to benchmark antibodies.

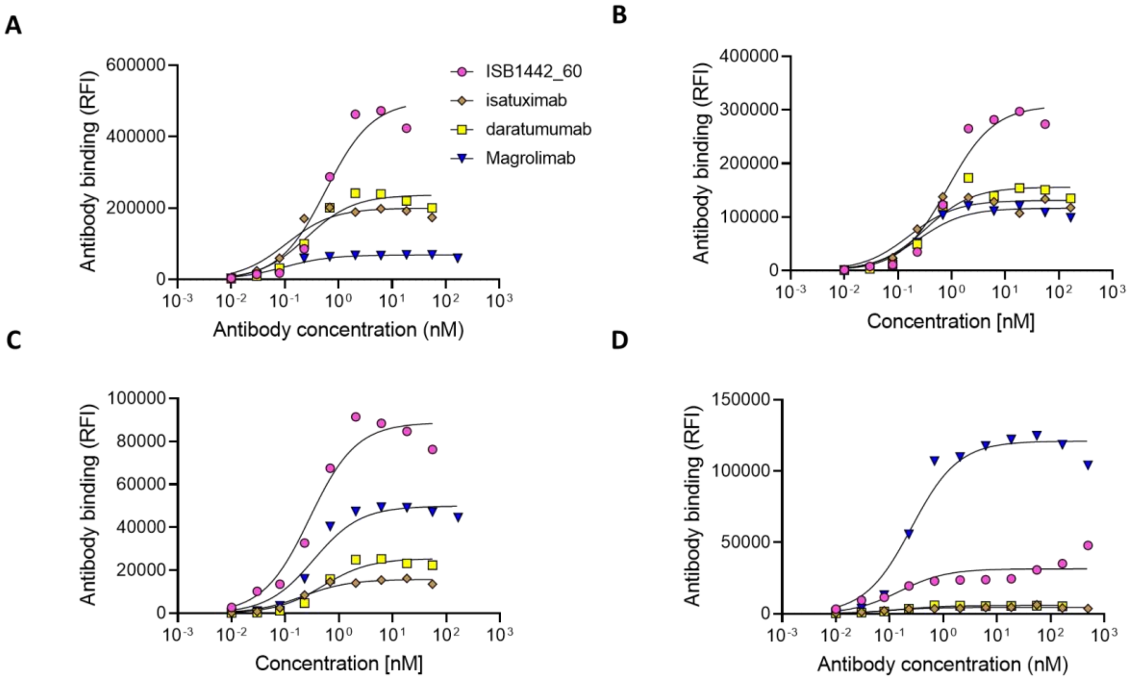

Supplementary Figure 5: ISB1442 is binding tumor cell lines expressing different level of CD38. (A: Daudi; B: Raji; C: NCI-H929; D: KMS-12-BM). A representative experiment of flow cytometric analyses of ISB1442 in comparison to anti-CD38 daratumumab or isatuximab and anti-CD47 (hu5F9) magrolimab on a panel of cell lines with different levels of CD38 and CD47 expression.

Supplementary Figure 6. Phagocytosis of CD38 high tumor cells

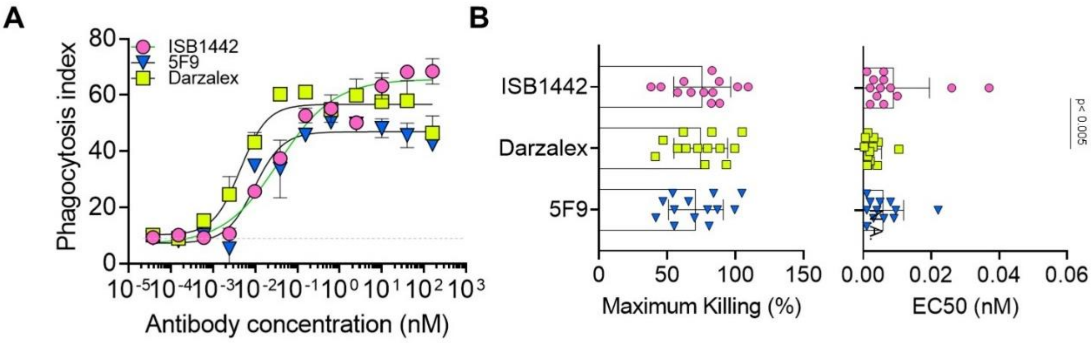

Supplementary Figure 6: Phagocytosis of CD38 high tumor cells

A. ADCP of CD38^{high} tumor cells induced by ISB 1442 or benchmark controls. A representative curve of specific phagocytosis induced by ISB1442 and benchmark molecules with CD38^{high} tumor cells (Daudi). B. Mean of duplicates is represented here +/- Standard Deviations. Compiled analyses of maximum phagocytosis and EC50 analyzed in multiple experiments with CD38^{high} tumor cells (Daudi). Each dot represents specific phagocytosis induced by ISB1442 with an independent macrophage donor. N=13. Statistics: paired T test.

Supplementary Figure 7. Live imaging of phagocytosis of CD38 high tumor cells

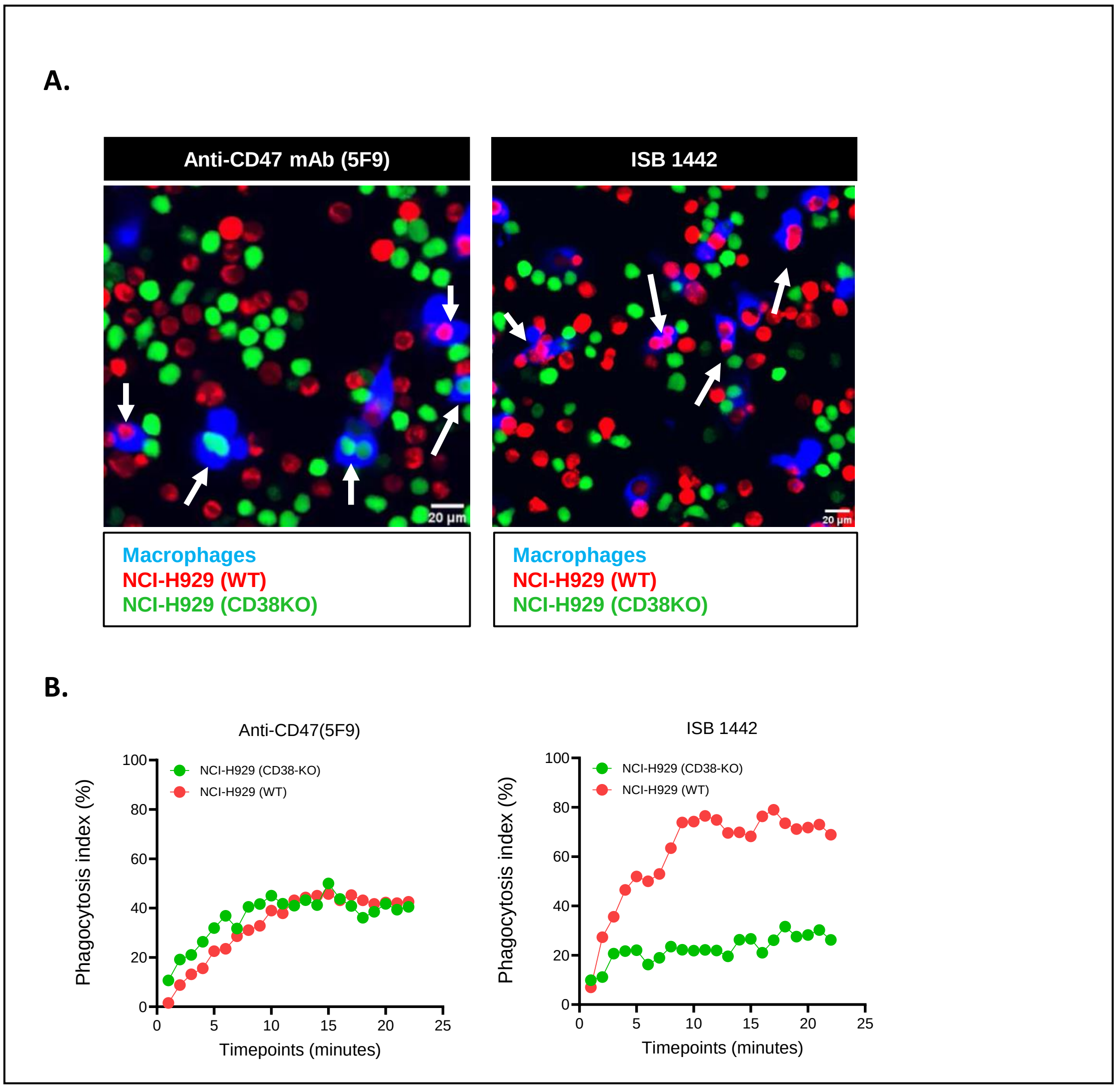

Supplementary Figure 7: ISB 1442-induced phagocytosis is CD38 specific. A. Snapshots from supplementary movies 3 and 4 showing monocytes-derived macrophages (blue) engulfing NCI-H929 CD38^{+} tumor cells (red) or NCI-H929 CD38-KO tumor cells (green). Arrows show examples of cells phagocytosis. B. Quantification of tumor cells phagocytosis as in A, performed by Perkin Elmer using a dedicated script from supplementary movie 2. N=1.

Supplementary Figure 8. Potency of ISB 1442 in concomitant treatment with daratumumab

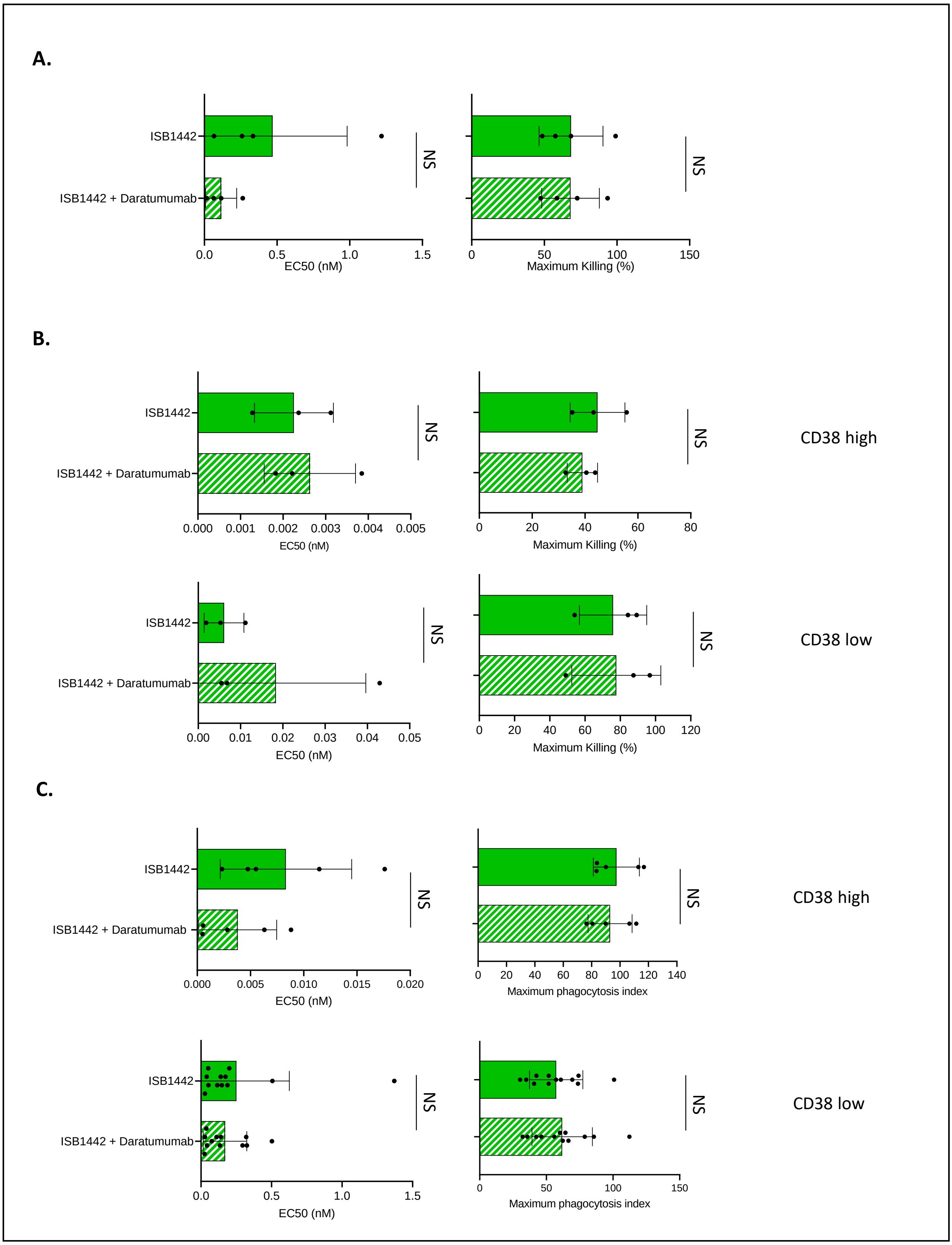

Supplementary Figure 8: ISB 1442 potency in presence of daratumumab.

Tumor cells were treated with equal quantity of daratumumab and ISB 1442 in all assay types. A. Compiled analyses of maximal killing and EC50 analyzed in multiple experiments in CD38^{high} (Daudi) tumor cells as

measured by CDC. Statistics: ordinary One Way Anova with Tukey's multiple comparison test. NS = Not Significant. N=4. B. Compiled analyses of maximal killing and EC50 analyzed in multiple experiments in CD38^{high} (Raji) and CD38^{low} (NCI-H929) tumor cells as measured by ADCC. Statistics: ordinary One Way Anova with Tukey's multiple comparison test. NS = Not Significant. N=3. C. Compiled analyses of maximal phagocytosis and EC50 analyzed in multiple experiments in CD38^{high} (Daudi) and CD38^{low} (KMS12-BM) tumor cells as measured by ADCP. Statistics: ordinary One Way Anova with Tukey's multiple comparison test. NS = Not Significant. N=11.

Supplementary Figure 9. Impact of CD38 and CD47 antigen sink on ISB 1442 potency

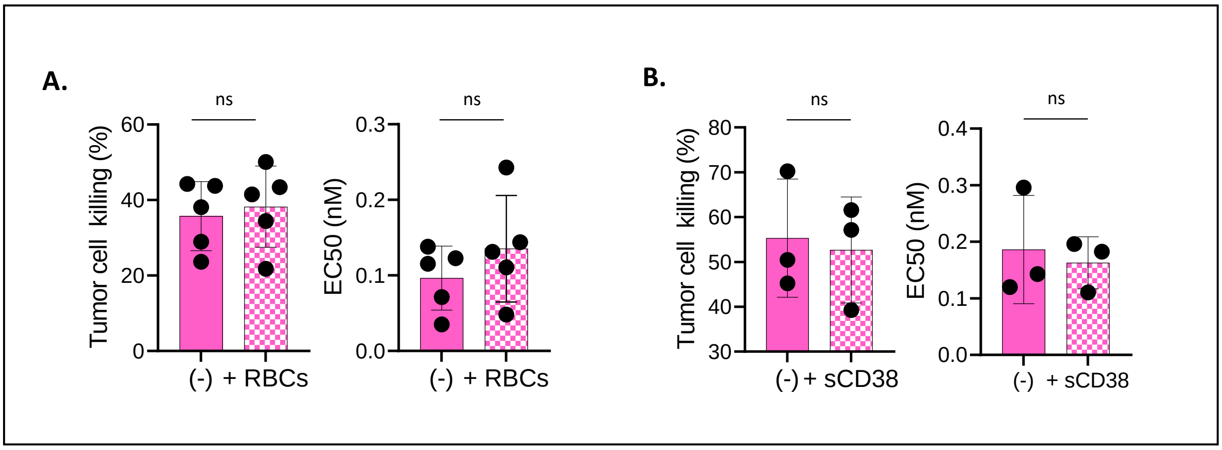

Supplementary Figure 9: ISB 1442 killing potency in presence of fresh human red blood cells or soluble CD38.

A. Compiled analyses of maximal killing (mean +/- SD) induced by ISB 1442 and benchmark antibodies in MMOAK using $CD38^{int}$ multiple myeloma cells (NCI-H929), in presence of fresh human RBCs (0.5M/well). A total of 5 PBMC/MDM donors have been evaluated in 2 independent experiments (n=5). Statistics, paired t test. ns: not significant. B. Compiled analyses of maximal killing (mean +/- SD) induced by ISB 1442 and benchmark antibodies in MMOAK using $CD38^{int}$ multiple myeloma cells (NCI-H929), in presence of 2.8ng/ml sCD38. A total of 5 PBMC/MDM donors have been evaluated in 2 independent experiments (n=5). Statistics, paired t test. ns: not significant

Supplementary Figure 10. On target on RBC and platelet: Hemolysis and platelet aggregation

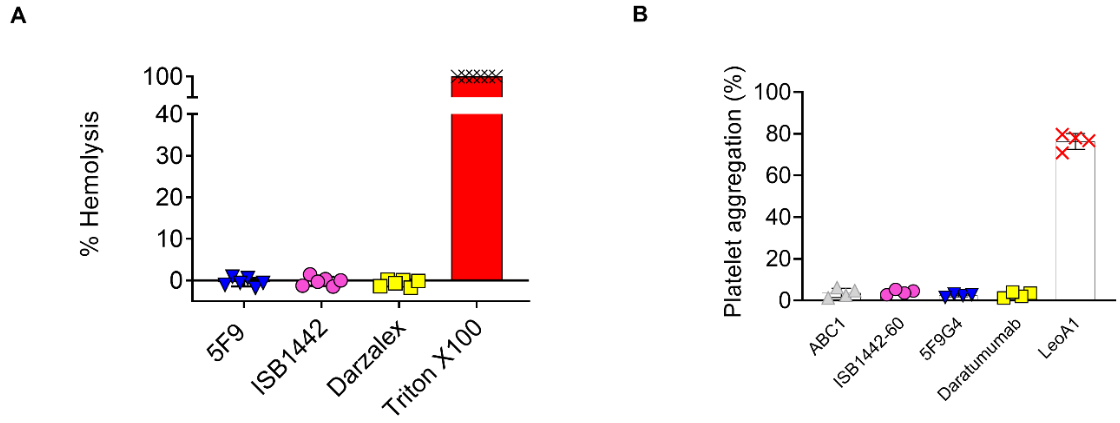

Supplementary Figure 10: Hemolysis or platelet aggregation analysis upon ISB 1442 treatment ex vivo.

A. Hemolysis of human peripheral blood in comparison to Triton X-100 used as a positive control (n= 6 healthy donors). B. Platelet aggregation of test antibodies as compared to positive control LeoA1 (n=4 healthy donors).

Supplementary Figure 11. FACS gating strategy

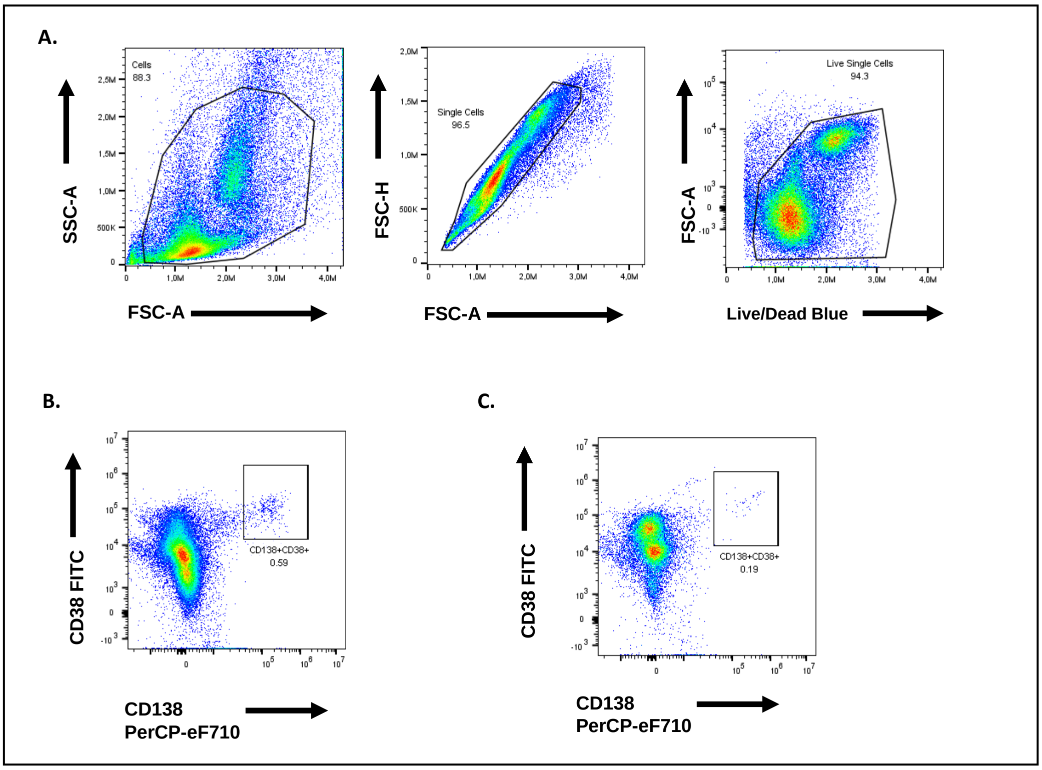

Supplementary Figure 11: FACS gating strategy.

A. Gating strategy used in flow cytometry experiments with primary MM samples. Live immune cell populations were first gated based on the FSC-A and SSC-A. Single cells were gated based on FSC-A and FSC-H and live cells based on their negative expression of live/dead blue viability staining. Plasma cells were further gated based on CD38 and CD138 expression. B-C Representative plot showing MM cells gating of an untreated patients' samples (B) or a sample treated with ISB 1442 at 100 nM (C).

Supplementary Table 1

	K_D to human protein (nM)	K_D to cynomolgus monkey protein (nM)
**CD47**	880 ± 80	940 ± 40
**CD38**	0.16 ± 0.01	0.48 ± 0.01

Table 1. Affinities of ISB 1442 in 2+1 bispecific format to human and cynomolgus monkey CD38 and CD47. Values are reported as mean ± standard deviation of at least three independent replicates.

Supplementary Table 2. Affinity to Human Fcγ Receptors by SPR

	Affinity to human Fc receptor (K_D, nM ± standard deviation)				Fold enhancement ISB 1442 over ISB 1442 WT Fc	Fold enhancement ISB 1442 over trastuzumab
	ISB 1442	ISB 1442 WT Fc	ISB 1442 silenced Fc	Trastuzumab		
**Human FcγRI**	0.02 ± 0.01	0.06 ± 0.01	No binding	0.21 ± 0.01	3.0	10.5
**Human FcγRIIA**	78 ± 7	224 ± 14	No binding	543 ± 25	2.9	7.0
**Human FcγRIIB**	315 ± 14	1140 ± 30	No binding	2520 ± 60	3.6	8.0
**Human FcγRIIIA**	13 ± 1	57 ± 2	No binding	142 ± 5	4.4	10.9
**Human FcRn**	181 ± 39	197 ± 40	221 ± 39	224 ± 21	1.1	1.2

Supplementary Table 2. Summary of K_D values measured by SPR for the interaction of ISB 1442, ISB 1442 WT Fc, ISB 1442 silenced Fc and trastuzumab with the different Fc receptors. Values are reported as mean ± standard deviation of at least three independent replicates.

Supplementary Table 3. Evaluation of CD38 and CD47 Expression in Tumor Cell Lines by sABC.

CD38 Expression on Cell Lines and human RBCs	CD38 sABC x10^3	CD47 sABC x10^3
Daudi (B cell lymphoma)	179 ± 51	66 ± 14
Raji (B cell lymphoma)	187 ± 46	125 ± 60
KMS-12-BM (MM)	11 ± 5	176 ± 26
NCI-H929 (PC; MM)	31 ± 35	94 ± 17

Supplementary Table 3: Absolute number of specific Antibody Bound per Cell (sABC) on multiple human cell lines. All the values are average (±Standard Deviation [SD]) from different affinity measurement experiments. The specific antibody binding capacity (sABC) indicates the relative target density.

Supplementary Table 4

	Cell Line	N	Cell affinity (nM) Mean +/- SD	Max Binding (RFI) Mean +/- SD
**CD38low**	**NCI-H929**	3	0.34 +/- 0.23	81x10^3 +/- 10 x10^3
	**KMS-12-BM**	4	0.14 +/- 0.03	29 x10^3 +/- 63 x10^2
**CD38high**	**Daudi**	4	0.9 +/- 0.43	44 x10^4 +/- 14 x10^4
	**Raji**	4	0.86 +/- 0.21	30 x10^4 +/- 12 x10^4

Supplementary Table 4: Cell affinity of ISB 1442 and maximal binding (Bmax) was evaluated by flow cytometry on a panel of CD38+ cell lines (Table 3). All the values are averages (±Standard Deviation [SD]) from different affinity measurement experiments. N = number of experiments.

Supplementary Table 5

Marker	Fluorochome	CLONE	Vendor
CD45	PerCP	2D1	biolegend
CD4	Spark YG581	SK3	biolegend
CD8	BUV661	SK1	BD
CD25	BV785	BC96	Biolegend
CD69	APC-eF780	FN50	Thermo
CD38	FITC	polyclonal	Cytognos/Med Tech
CD138	PerCP-eF710	MI15	Thermo
CD107a	BV605	H4A3	biolegend
CD56	V450	B159	BD
CD11b	APC	ICRF44	Biolegend
Live/Dead NIR			Thermofisher
CD47	eF450	B6H12	Thermo
BCMA	APC	19F2	Biolegend
CD138	BV605	281-2	Biolegend
CD38	FITC	polyclonal	Cytognos
CD45	PerCP-eF710	HI30	Thermo
CD45	PerCP	HI30	Thermo
CD11b	BV711	M1/70	Biolegend
CD56	PE-eF610	NCAM16.2	Thermo
CD25	PC7	BC96	biolegend
CD8	BV650	SK1	Biolegend
CD69	BV785	FN50	Biolegend
CD107a	AF700	H4A3	BD
BCMA (CD269)	PE-Dazzle 594	19F2	Biolegend
CD11b	APC-R700	M1/70	BD
CD11c	BUV805	B-ly6	BD
CD138	PerCP-eFluor 710	MI15	Thermo
CD172a	SB600	15-414	Thermo
CD25	PE-Fire700	M-A251	Biolegend
CD38	FITC	polyclonal	Cytognos/Med Tech
CD4	cfluor YG584	SK3	Cytek
CD45	PerCP	2D1	Biolegend
CD47	APC-Fire750	CC2C6	Biolegend
CD56	BUV737	NCAM16.2	BD
CD8	cFluor V547	SK1	Cytek
CD20	BUV395	2H7	BD biosciences
HLA-DR	BUV496	Tu39	BD biosciences
CD16	BUV615	3G8	BD biosciences
CD19	eF450	HIB19	ThermoFisher
CD69	BV650	FN50	Biolegend
NKp30 (CD337)	BV785	P30-15	Biolegend
CD107a	APC	H4A3	Biolegend

Supplementary Table 5: Information on antibodies used for flow cytometry experiments.

Supplementary Movie 1: Live imaging of phagocytosis of CD38 positive tumor cells

Live imaging performed with Perkin Elmer Operetta in the presence of control antibody. Monocytes-derived macrophages (blue) engulfing of KMS-12-BM $CD38^{low}$ tumor cells (green).

Supplementary Movie 2: Live imaging of phagocytosis of CD38 positive tumor cells

Live imaging performed with Perkin Elmer Operetta in the presence of ISB1442 used at 80nM. Monocytes-derived macrophages (blue) engulfing of KMS-12-BM $CD38^{low}$ tumor cells (green).

Supplementary Movie 3: Live imaging of phagocytosis of CD38-KO tumor cells

Live imaging performed with Perkin Elmer Operetta in the presence of anti-CD47 (5F9) used at 8nM. Monocytes-derived macrophages (blue) engulfing NCI-H929 $CD38^{+}$ tumor cells (red) or NCI-H929 CD38-KO tumor cells (green).

Supplementary Movie 4: Live imaging of phagocytosis of CD38-KO tumor cells

Live imaging performed with Perkin Elmer Operetta in the presence of ISB1442 used at 8nM. Monocytes-derived macrophages (blue) engulfing NCI-H929 $CD38^{+}$ tumor cells (red) or NCI-H929 CD38-KO tumor cells (green).
