# Supplementary material for: Development of ISB 1442, a CD38 and CD47 bispecific biparatopic antibody innate cell modulator for the treatment of multiple myeloma
Source: Nat Commun. 2024 Mar 6;15:2054. doi: 10.1038/s41467-024-46310-y (PMC10917784; doi:10.1038/s41467-024-46310-y)
Supplement: Supplementary file 3 — Description of Additional Supplementary Files [file 41467_2024_46310_MOESM3_ESM.pdf]

Description of Additional Supplementary Files

File Name: Supplementary Movie 1
Description: **Live imaging of phagocytosis of CD38 positive tumor cells**
Live imaging performed with Perkin Elmer Operetta in the presence of control antibody. Monocytes-derived macrophages (blue) engulfing of KMS-12-BM $CD38^{low}$ tumor cells (green).

File Name: Supplementary Movie 2
Description: **Live imaging of phagocytosis of CD38 positive tumor cells**
Live imaging performed with Perkin Elmer Operetta in the presence of ISB1442 used at 80nM. Monocytes-derived macrophages (blue) engulfing of KMS-12-BM $CD38^{low}$ tumor cells (green).

File Name: Supplementary Movie 3
Description: **Live imaging of phagocytosis of CD38-KO positive tumor cells**
Live imaging performed with Perkin Elmer Operetta in the presence of anti-CD47 (5F9) used at 8nM. Monocytes-derived macrophages (blue) engulfing NCI-H929 $CD38^{+}$ tumor cells (red) or NCI-H929 CD38-KO tumor cells (green).

File Name: Supplementary Movie 4
Description: **Live imaging of phagocytosis of CD38-KO positive tumor cells**
Live imaging performed with Perkin Elmer Operetta in the presence of ISB1442 used at 8nM. Monocytes-derived macrophages (blue) engulfing NCI-H929 $CD38^{+}$ tumor cells (red) or NCI-H929 CD38-KO tumor cells (green).
