# Supplementary material for: Development of ISB 1442, a CD38 and CD47 bispecific biparatopic antibody innate cell modulator for the treatment of multiple myeloma
Source: Nat Commun. 2024 Mar 6;15:2054. doi: 10.1038/s41467-024-46310-y (PMC10917784; doi:10.1038/s41467-024-46310-y)
Supplement: Supplementary file 8 — Reporting Summary [file 41467_2024_46310_MOESM8_ESM.pdf]

nature portfolio

Corresponding author(s): Mario Perro, Stefano Sammicheli

Last updated by author(s): Feb 16, 2024

Reporting Summary

Nature Portfolio wishes to improve the reproducibility of the work that we publish. This form provides structure for consistency and transparency in reporting. For further information on Nature Portfolio policies, see our Editorial Policies and the Editorial Policy Checklist.

Statistics

For all statistical analyses, confirm that the following items are present in the figure legend, table legend, main text, or Methods section.

n/a	Confirmed	
☐	☒	The exact sample size (*n*) for each experimental group/condition, given as a discrete number and unit of measurement
☐	☒	A statement on whether measurements were taken from distinct samples or whether the same sample was measured repeatedly
☐	☒	The statistical test(s) used AND whether they are one- or two-sided<br>*Only common tests should be described solely by name; describe more complex techniques in the Methods section.*
☒	☐	A description of all covariates tested
☐	☒	A description of any assumptions or corrections, such as tests of normality and adjustment for multiple comparisons
☐	☒	A full description of the statistical parameters including central tendency (e.g. means) or other basic estimates (e.g. regression coefficient) AND variation (e.g. standard deviation) or associated estimates of uncertainty (e.g. confidence intervals)
☐	☒	For null hypothesis testing, the test statistic (e.g. *F*, *t*, *r*) with confidence intervals, effect sizes, degrees of freedom and *P* value noted<br>*Give P values as exact values whenever suitable.*
☒	☐	For Bayesian analysis, information on the choice of priors and Markov chain Monte Carlo settings
☒	☐	For hierarchical and complex designs, identification of the appropriate level for tests and full reporting of outcomes
☒	☐	Estimates of effect sizes (e.g. Cohen's *d*, Pearson's *r*), indicating how they were calculated

Our web collection on statistics for biologists contains articles on many of the points above.

Software and code

Policy information about availability of computer code

Data collection	Biacore 8K+ instrument (Cytiva)<br>OctetRED96e instrument (Sartorius)<br>CellInsight CX5 High Content Screening Platform (Thermofisher)<br>Operetta (Perkin Elmer)<br>AggRAM (Helena Biosciences)<br>Cell counter Logos Biosystems LUNA-FL™ Automated Fluorescence Cell Counter<br>Flow cytometer Beckman Coulter Cytoflex<br>Flow Cytometer Sartorius iQue Screener Plus<br>High-Content Screening-Analyzer Thermofisher CellInsight CX5<br>Plate Reader BioTek Synergy Neo plate reader<br>Hematology analyzer (human blood) Swissavans Sigma 5H<br>Hematology analyzer (animal blood) Swissavans Sigma5V<br>Centrifuge for ID-cards Biorad ID-centrifuge12SII<br>FACSDiva
Data analysis	GraphPad Prism v. 9.0; FlowJo, v10.7.2, CytExpert 2.5.0.77, PyMol v2.5, Biacore Insight Evaluation software v2.0, Octet Data Analysis HT 11.1

For manuscripts utilizing custom algorithms or software that are central to the research but not yet described in published literature, software must be made available to editors and reviewers. We strongly encourage code deposition in a community repository (e.g. GitHub). See the Nature Portfolio guidelines for submitting code & software for further information.

Data

Policy information about availability of data

All manuscripts must include a data availability statement. This statement should provide the following information, where applicable:

- Accession codes, unique identifiers, or web links for publicly available datasets
- A description of any restrictions on data availability
- For clinical datasets or third party data, please ensure that the statement adheres to our policy

The data supporting the findings of this study are available within the article and its supplementary Information files and from the corresponding authors upon reasonable request

Research involving human participants, their data, or biological material

Policy information about studies with human participants or human data. See also policy information about sex, gender (identity/presentation), and sexual orientation and race, ethnicity and racism.

Reporting on sex and gender	Due to limited availability of fresh primary patients' material. samples were used as they became available and priority was given to different treatment history rather the the gender of each patients.
Reporting on race, ethnicity, or other socially relevant groupings	The findings in this study were not involved in race, ethnicity, or other socially relevant grouping.
Population characteristics	Blood samples were derived from healthy donors. Multiple myeloma samples were obtained from academic collaborators as stated in M&M section.
Recruitment	Healthy donors were recruited by Transfusion Interregionale CRS (Lausanne, Switzerland). Bone-Marrow Aspirates or peripheral blood samples from multiple myeloma patients were obtained from University Hospital Geneva, Centre Hospitalier Universitarie Nantes (MYRACLE cohort 42) and from University Oxford Hospitals with informed consent under each site ethical approvals
Ethics oversight	All research on healthy human donor was approved by transfusion Interregionale CRS, with all donors provided written informed consent in accordance with the Declaration of Helsinki and the protocol of the local institutional review board, the Medical Ethics Committee of Transfusion Interregionale CRS.<br>All research on multiple myeloma patient's samples were performed in accordance with ethical approvals with collaborating institutes. Oxford Clinical Research: The study was approved by the Oxford Clinical Research Ethics Committee (17/SC/0572) and the HaemBiobank Governance Committee (BBProj-27.0 and BBProj-13.0). Nantes: all samples were obtained from the cohort MYRACLE (Benanina L et al., BMC Cancer, 2019), NCT03807128. University Hospital Geneve (HUG): ethical approval number 2021-02416.

Note that full information on the approval of the study protocol must also be provided in the manuscript.

Field-specific reporting

Please select the one below that is the best fit for your research. If you are not sure, read the appropriate sections before making your selection.

☒ Life sciences ☐ Behavioural & social sciences ☐ Ecological, evolutionary & environmental sciences

For a reference copy of the document with all sections, see nature.com/documents/nr-reporting-summary-flat.pdf

Life sciences study design

All studies must disclose on these points even when the disclosure is negative.

Sample size	No statistical test was used to determined sample size. Instead sample size was determined empirically according to previous knowledge of the variation in experimental setup
Data exclusions	For potency testing in vitro, two exclusion criteria were used before EC50 extraction assessing the shape of the curve leading to the inclusion of regular pharmacological dose-dependent fit: R2 superior to 0.7, to ensure the quality of the fit (regression used is sigmoidal dose response, GraphPad Prism software) and Span (difference between top fit and bottom fit) superior to 10% (window of response)
Replication	With the exception of imaging experiments, only data that we were able to replicate at least in two independent experiments were included in the manuscript
Randomization	For all in vivo experiments mice were randomized by tumor volume to first achieve the same average starting tumor volume then a similar standard deviation of tumor volumes between all groups. In the experiment reported in 4 c,d,e outliers (by tumor volume) were excluded at randomization to better match standard deviations between groups.

Blinding	In figure 4 a,b technicians were blinded to treatment groups. In 4 c,d,e blinding was not possible and blinding was not conducted for 4f as treatment effects were not the outcome under evaluation.

Reporting for specific materials, systems and methods

We require information from authors about some types of materials, experimental systems and methods used in many studies. Here, indicate whether each material, system or method listed is relevant to your study. If you are not sure if a list item applies to your research, read the appropriate section before selecting a response.

Materials & experimental systems

n/a	Involved in the study
☐	☒ Antibodies
☐	☒ Eukaryotic cell lines
☒	☐ Palaeontology and archaeology
☐	☒ Animals and other organisms
☒	☐ Clinical data
☒	☐ Dual use research of concern
☒	☐ Plants

Methods

n/a	Involved in the study
☒	☐ ChIP-seq
☐	☒ Flow cytometry
☒	☐ MRI-based neuroimaging

Antibodies

Antibodies used	Produced in house: anti-CD38-B6 Fab, anti-CD38-E2 Fab, anti-CD38-E2-RecA Fab, anti-CD38-B6-D9 Fab, anti-CD7-H2 Fab, daratumumab Fab, hu5F9 Fab, ISB 1442 and all variants and control molecules described in Figure 2, trastuzumab.<br>Purchased: daratumumab (Janssen)<br>Antibodies used for figure 6 (potency ex vivo on patients samples), including full information on clones, fluorochromes and vendors are included in a table in M&M section.<br>For quantification of CD47 (sABC) on tumor cells, monoclonal anti-CD47 (5F9) was used and produced in house (see M&M section "Antibodies/Treatment").<br>For quantification of CD38 (sABC) on tumor cells, monoclonal anti-CD38 (daratumumab) was purchased from Janssen Biotech Inc.
Validation	All antibodies were titrated with a dose-response from at least 1/50 with a serial dilution of 1/2 up to 1/3200 on positive cells and non-expressing cells. Choice of the optimal antibody dilution is based on the stain index calculation Stain Index (SI)=(MFI of positive population -MFI of negative population)/(2*SD of negative population)

Eukaryotic cell lines

Policy information about cell lines and Sex and Gender in Research

Cell line source(s)	Raji cell line was purchased from ATCC. KMS-12-BM, NCI-H929, MOLP8 and Daudi were obtained from DSMZ. Raji-KO and NCI-H929 KO cell lines have been generated in-house using CRISPR/CAS9 technology.<br>HEK293-EBNA cells (ATCC), CHO-S (Invitrogen).
Authentication	Authentication was performed using short tandem repeat (STR) analysis evaluated by by Microsynth (Balgach, Switzerland) at passage 5 and passage 15 according to Microsynth guidelines
Mycoplasma contamination	All cell lines were tested negative for mycoplasma
Commonly misidentified lines<br>(See ICLAC register)	No commonly misidentified cell lines were used (according to ICLAC register version 10)

Animals and other research organisms

Policy information about studies involving animals; ARRIVE guidelines recommended for reporting animal research, and Sex and Gender in Research

Laboratory animals	CB-17/Icr-Prkdcscid/scid/Rj mice were used for all tumor studies. All animals were females 6-8 weeks of age at study start with a body weight of 18-22g. Mice were housed in an SPF facility for all animal experiments. CBySmn.Cg-Prkdcscid/J mice (catalog #001803) were used for all PK studies. Female mice were used at 6-8 weeks of age and 20+g body weight.All mice were sourced from Janvier Labs (catalog: CB-17/Icr-Prkdcscid/scid/Rj) Note: Janvier uses the mouse strain name as the catalog number.
Wild animals	No wild animals were used
Reporting on sex	Female animals were used for these studies due to ethical concerns around randomization in cancer studies. Female mice of these strains could be grouped together post randomization for each treatment ensuring reduced risk of technical error in dosing (if mice from two or more groups were in the same cage) without requiring single housing of mice. This respected the 3Rs by minimizing both the risk of waste of animal life through technical error and the elevated stress of experimentation by single housing mice.

Field-collected samples	No field collection occured
Ethics oversight	Ethical oversight of all animal work conducted at Ichnos sciences (figure 4c,d,e) was through Swiss federal and Cantonal oversight of animal experimentation. All research was approved under Swiss federal license number 33147. Figure 4f was conducted under the ethical oversight of the Jackson Laboratories IACUC (Bar Harbor, Maine, USA). Data reported in figure 4a,b was conducted under the ethical oversight of Transcure (Archamps, France) IACUC. Both Transcure and Jackson laboratories are AAALAC accredited.

Note that full information on the approval of the study protocol must also be provided in the manuscript.

Flow Cytometry

Plots

Confirm that:

☐ The axis labels state the marker and fluorochrome used (e.g. CD4-FITC).

☐ The axis scales are clearly visible. Include numbers along axes only for bottom left plot of group (a 'group' is an analysis of identical markers).

☐ All plots are contour plots with outliers or pseudocolor plots.

☐ A numerical value for number of cells or percentage (with statistics) is provided.

Methodology

Sample preparation	Sample preparation is described in details in the M&M section of the manuscript in the flow cytometry assays section. For quantification of surface antibodies bound to cells (ABC), expression levels of CD38 and CD47 were determined using Qifikit® (for mouse primary antibodies) or Biocytex® kit (for human primary antibodies) according to the manufacturer's instructions. This method allows the quantification of antigenic sites on cell surfaces by providing an ABC value which corresponds to the number of primary mouse or human monoclonal antibodies bound per cell. A specific ABC value was derived after background correction (isotype control-induced background). Briefly, one hundred thousand cells were plated in each well of a U-bottom 96-well plate (TPP) and incubated with saturating concentrations of mouse or human primary antibodies for 30 min at 4°C. After two washing steps, cells and calibration beads were stained in parallel with a FITC-anti mouse IgG (for Qifikit®) or a FITC-anti-human IgG (Biocytex® kit) according to manufacturer's instructions for 30min at 4°C. After two last washing steps, the samples were suspended in PBS containing 2.5% FCS 2mM EDTA and 0.05% NaN3 and a viability dye. (Dapi or Sytox). Samples were acquired on a Cytoflex cytometer (Beckman Coulter) and data were analyzed with Flow cytometry software (FlowJo, BD). A calibration curve was constructed by plotting the mean fluorescence intensity (MFI) of the individual bead populations against the number of defined antibody molecules on the beads. The number of primary antibodies bound on the cells (sABC value) was then determined by interpolation using this calibration curve.
Instrument	Cytoflex LX Flow Cytometer (Beckman Coulter; ref no. C40321)<br>FACSFortessa Flow Cytometer (BD; ref no. 67465)
Software	FlowJo, v10.7.2
Cell population abundance	Monocytes and NK cells purities after isolation at the start of assay > 95%
Gating strategy	Figure 3 E-J, ADCC and MMoAK assays: gating strategy described in M&M, section "Antibody-Dependent-Cell-Cytotoxicity Assay and MMoAK assay"<br>Figure 6: FSC/SSC plot was used to gate cells and exclude debris; FSC-A/FSC-H to gate single cells; Live cells: FSC-A vs Live Dead Blue; CD138 vs CD38 on single live cells to identify plasma cells

☒ Tick this box to confirm that a figure exemplifying the gating strategy is provided in the Supplementary Information.
